# Distinct acute effects of LSD, MDMA, and d-amphetamine in healthy subjects

**DOI:** 10.1038/s41386-019-0569-3

**Published:** 2019-11-16

**Authors:** Friederike Holze, Patrick Vizeli, Felix Müller, Laura Ley, Raoul Duerig, Nimmy Varghese, Anne Eckert, Stefan Borgwardt, Matthias E. Liechti

**Affiliations:** 1 Department of Biomedicine and Department of Clinical Research, Division of Clinical Pharmacology and Toxicology, University Hospital Basel, University of Basel, Basel, 4056 Switzerland; 2 Psychiatric University Hospital (UPK), University of Basel, Basel, 4012 Switzerland; 3 Transfaculty Research Platform Molecular and Cognitive Neuroscience, University of Basel, Basel, Switzerland

**Keywords:** Human behaviour, Pharmacology, Human behaviour, Pharmacology

## Abstract

Lysergic acid diethylamide (LSD) is a classic psychedelic, 3,4-methylenedioxymethamphetamine (MDMA) is an empathogen, and d-amphetamine is a classic stimulant. All three substances are used recreationally. LSD and MDMA are being investigated as medications to assist psychotherapy, and d-amphetamine is used for the treatment of attention-deficit/hyperactivity disorder. All three substances induce distinct acute subjective effects. However, differences in acute responses to these prototypical psychoactive substances have not been characterized in a controlled study. We investigated the acute autonomic, subjective, and endocrine effects of single doses of LSD (0.1 mg), MDMA (125 mg), d-amphetamine (40 mg), and placebo in a randomized, double-blind, cross-over study in 28 healthy subjects. All of the substances produced comparable increases in hemodynamic effects, body temperature, and pupil size, indicating equivalent autonomic responses at the doses used. LSD and MDMA increased heart rate more than d-amphetamine, and d-amphetamine increased blood pressure more than LSD and MDMA. LSD induced significantly higher ratings on the 5 Dimensions of Altered States of Consciousness scale and Mystical Experience Questionnaire than MDMA and d-amphetamine. LSD also produced greater subjective drug effects, ego dissolution, introversion, emotional excitation, anxiety, and inactivity than MDMA and d-amphetamine. LSD also induced greater impairments in subjective ratings of concentration, sense of time, and speed of thinking compared with MDMA and d-amphetamine. MDMA produced greater ratings of good drug effects, liking, high, and ego dissolution compared with d-amphetamine. d-Amphetamine increased ratings of activity and concentration compared with LSD. MDMA but not LSD or d-amphetamine increased plasma concentrations of oxytocin. None of the substances altered plasma concentrations of brain-derived neurotrophic factor. These results indicate clearly distinct acute effects of LSD, MDMA, and d-amphetamine and may assist the dose-finding in substance-assisted psychotherapy research.

## Introduction

Lysergic acid diethylamide (LSD) is a classic serotonergic hallucinogen that has been widely used recreationally [1] and to a limited extent in psychiatric research [2]. LSD acutely induces marked alterations of waking consciousness [3] that have been shown to primarily depend on an interaction with the serotonin 5-hydroxytryptamine-2A (5-HT_2A_) receptor [4], although LSD also acts on 5-HT_1_ and dopamine receptors [5]. Recent clinical trials indicate that the quality of the acute psychedelic experience in response to psilocybin or LSD predicts long-term changes in mental health and well-being in patients and healthy persons [6–11]. For example, greater psilocybin-induced mystical-type experiences and more pronounced and more positive acute alterations of consciousness were associated with lasting antidepressant responses in patients with depression [6, 7]. 3,4-Methylenedioxymethamphetamine (MDMA) is the active compound in the recreational substance ecstasy and is currently investigated as an adjunct to psychotherapy to treat post-traumatic stress disorder (PTSD) [12, 13]. MDMA not only exhibits some amphetamine-like properties but also shows hallucinogenic-like effects and can be considered an intermediate substance between a pure stimulant like d-amphetamine and a pure hallucinogenic drug like LSD. MDMA acutely induces feelings of well-being, love, empathy, and prosociality [14, 15], and produces mild perceptual alterations that are thought to be primarily mediated by the release of serotonin (5-HT) [16, 17] and norepinephrine [18], and the direct activation of 5-HT_2A_ receptors [19]. Additionally, MDMA releases oxytocin [14, 20, 21], which may contribute to the mediation of its prosocial effects [22, 23]. The unique emotional effects of MDMA lead to its classification as an empathogen or entactogen [24], referring to assumingly distinct effects from psychostimulants [25–28]. Psychostimulants such as d-amphetamine and methamphetamine primarily activate dopamine and norepinephrine systems, with only minimal effects on 5-HT [29, 30], and promote stimulation, wakefulness, and concentration without the MDMA-typical emotional effects [25, 27, 28, 31–35]. Although MDMA produces less profound changes in perception compared with classic hallucinogens, it is often also classified as a psychedelic substance. On the other hand, LSD was found to exhibit MDMA-like empathogenic mood effects such as increased closeness, openness, and trust [3], indicating overlapping properties with MDMA [14, 27] potentially useful to assist psychotherapy. Whether and how the effects of MDMA are similar or differ from the classic stimulant d-amphetamine and classic hallucinogen LSD have not been studied under double-blind conditions in the same study. Comparative studies, particularly within-subjects comparisons of the acute effects of these prototypical substances, are lacking. Therefore, we compared for the first time the acute subjective, autonomic, and endocrine effects of doses with similar cardiovascular activity (“equivalent” doses) of LSD (0.1 mg), MDMA (125 mg), d-amphetamine (40 mg), and placebo in a cross-over study in healthy subjects. By comparing all three substances using a within-subject design, it is possible to directly assess differences and commonalities of these substances. Moreover, by including different substances with partially overlapping effects, it is also possible to considerably improve blinding. This latter point has been a serious shortcoming of almost all previous studies, which compared effects of MDMA and LSD, respectively, with non-active placebo, which almost inevitably results in unblinding. Dose selection was critical because we could only compare single doses of each substance in this within-subjects study. LSD was used at an intermediate dose of 0.1 mg that is representative of doses that are used recreationally [36] and in research [2]. A higher dose of 0.2 mg LSD has previously been shown to produce greater subjective effects than the 0.1 mg dose [37, 38], but was not used in the present study because it was expected to produce greater alterations of waking consciousness than any of the other substances and would not have allowed brain imaging due to expected anxiety and movement artifacts in the scanner. MDMA was used at a high dose (125 mg) that produces the full range of empathogenic MDMA-typical effects [27] and is considered safe [39], and at the upper range of doses used in research investigating the safety and efficacy of MDMA-assisted psychotherapy in the treatment of PTSD [12] and in experimental studies in healthy participants [27, 39, 40]. Preferred recreational doses are slightly lower and in the range of 80–120 mg [41]. Higher doses are expected to produce largely similar subjective positive responses, but considerably more adverse effects [39, 41]. d-Amphetamine was also used at a rather high dose (40 mg) that is in the upper range of doses that are used in patients and in research [31, 32, 34, 42–44].

The main goal of the present study was to describe and compare the subjective and autonomic effects of all three substances over time and determine plasma concentration–time profiles (pharmacokinetics). We hypothesized that LSD would induce more pronounced and different alterations of waking consciousness, assessed by the 5 Dimensions of Altered States of Consciousness (5D-ASC) scale and Mystical Experience Questionnaire (MEQ) compared with MDMA and d-amphetamine [37]. We predicted that MDMA would produce distinct subjective emotional effects compared with d-amphetamine [25, 27, 28] and induce greater increases in plasma concentrations of oxytocin than LSD and d-amphetamine [3, 14]. Finally, we explored effects on plasma concentrations of brain-derived neurotrophic factor (BDNF), a biomarker that is linked to neurogenesis, because psychedelics have been shown to have neuroregenerative potential and may alter BDNF [45, 46]. Altogether, we tested whether prototypical hallucinogens, empathogens, and psychostimulants are indeed substances with distinct acute-effect profiles in humans for the first time using a head-to-head comparison with the same study and participants.

## Materials and methods

### Study design

We used a double-blind, placebo-controlled, cross-over design with four experimental test sessions to investigate the responses to 0.1 mg LSD, 125 mg MDMA, 40 mg d-amphetamine, and placebo in 28 healthy participants (14 females, 14 males). The washout period between sessions was at least 10 days. The study was conducted in accordance with the Declaration of Helsinki and approved by the Ethics Committee northwest Switzerland (EKNZ). The administration of LSD, MDMA, and d-amphetamine in healthy subjects was authorized by the Swiss Federal Office for Public Health, Bern, Switzerland. All of the participants provided written consent before participating in the study, and they were paid for their participation. The study was registered at ClinicalTrials.gov (NCT03019822).

### Participants

Twenty-eight healthy subjects (14 men, 14 women; 28 ± 4 years old [mean ± SD]; range, 25–45 years; body weight, 71.5 ± 12.0 kg) were recruited from the University of Basel. Participants who were younger than 25 years old were excluded from participating in the study because of the higher incidence of psychotic disorders and because low age has been associated with more anxious reactions to hallucinogens [47]. Additional exclusion criteria were age >50 years, pregnancy (urine pregnancy test at screening and before each test session), personal or family (first-degree relative) history of major psychiatric disorders (assessed by the Semi-structured Clinical Interview for Diagnostic and Statistical Manual of Mental Disorders, 4th edition, Axis I disorders by a trained psychiatrist), the use of medications that may interfere with the study medications (e.g. antidepressants, antipsychotics, sedatives), chronic or acute physical illness (abnormal physical exam, electrocardiogram, or hematological and chemical blood analyses), tobacco smoking (>10 cigarettes/day), lifetime prevalence of illicit drug use >10 times (except for Δ^9^-tetrahydrocannabinol), illicit drug use within the last 2 months, and illicit drug use during the study (determined by urine drug tests). A previous study found no difference in the response to LSD between hallucinogen‐naive and moderately experienced subjects (<10 times) [3]. However, we wanted to exclude frequent substance users because extensive previous uncontrolled experiences may influence/condition new substance experiences [47]. The participants were asked to abstain from excessive alcohol consumption between test sessions (no more than 10 standard drinks/week) and particularly limit their use to one drink on the day before the test sessions. Additionally, the participants were not allowed to drink xanthine-containing liquids after midnight before the study day.

Five participants had previously used a hallucinogen, including LSD (three participants, 1–4 times), DMT (one participant 4 times), and salvia divinorum (one participant 3 times), eight participants had used MDMA (1–5 times), and 13 participants had previously used a stimulant, including methylphenidate (six participants, 1–3 times), amphetamine (eight participants, 1–2 times), and cocaine (one participant, 4 times). Eight participants had never used any illicit drugs with the exception of cannabis.

We performed urine drug tests at screening and before each test session, and no substances were detected during the study. We did not screen for alcohol use.

### Study procedures

The study included a screening visit, a psychiatric interview, four 12-h experimental sessions, and an end-of-study visit. The experimental sessions were conducted in a quiet standard hospital patient room. Only one research subject and one investigator were present during the experimental sessions. The participants could interact with the investigator, rest quietly, or listen to music via headphones, but no other entertainment was provided. LSD, d-amphetamine, or placebo was administered at 9:00 a.m. MDMA or placebo was administered at 9:30 a.m. This was because of the different times to peak effects for each substance so that the functional magnetic resonance imaging (fMRI) scan and other assessments could be performed during the expected time-matched peak drug effects [26, 27, 32, 48, 49]. The fMRI scan was performed at 11:00 a.m.–12:00 p.m. and the fMRI findings will be published elsewhere. Autonomic and subjective effects were assessed repeatedly throughout the session. Blood was collected to determine endocrine effects and substance concentrations.

### Study drugs

LSD (D-lysergic acid diethylamide base, high-performance liquid chromatography purity >99%; Lipomed AG, Arlesheim, Switzerland) was administered in a single intermediate oral dose of 100 µg [50]. d-Amphetamine sulfate (40 mg salt; Hänseler, Herisau, Switzerland) was administered in a relatively high dose in the form of gelatin capsules as a single oral dose that corresponded to 30 mg d-amphetamine base [32]. MDMA hydrochloride (Lipomed AG, Arlesheim, Switzerland) was prepared as gelatin capsules and administered as a single oral dose of 125 mg, which is considered a relatively high dose [28, 40, 51, 52]. Blinding to treatment was guaranteed by using a double-dummy method, with identical capsules and vials that were filled with mannitol and ethanol, respectively, as placebo. At the end of each session and at the end of the study, the participants were asked to retrospectively guess their treatment assignment.

### Measures

#### Subjective effects

Subjective effects were assessed repeatedly using visual analog scales (VASs) 1 and 0.5 h before and 0, 0.5, 1, 1.5, 2.5, 3, 4, 5, 6, 7, 8, 9, 10, and 11 h after drug administration (time specifications correspond to MDMA administration). The VASs included “any drug effect,” “good drug effect,” “bad drug effect,” “drug liking,” “drug high,” “stimulated,” “ego dissolution,” “talkative,” “open,” “concentration,” “sense of time,” and “speed of thinking” [14]. The VASs were presented as 100-mm horizontal lines (0–100%), marked from “not at all” on the left to “extremely” on the right (“slowed” and “racing” for “speed of thinking”). The VASs for “open,” “talkative,” “concentration,” “sense of time,” and “speed of thinking” were bidirectional (±50%), marked from “not at all” on the left (−50) to “normal” in the middle (0) and to “extremely” on the right (+50). The 60-item Adjective Mood Rating Scale (AMRS) [53] was administered 1 h before and 1.5, 4, and 11 h after drug administration. The 5D-ASC scale [54, 55] was administered 11 h after drug administration to retrospectively rate alterations in waking consciousness induced by the drugs. Mystical experiences were assessed using the German version [37] of the 100-item States of Consciousness Questionnaire [56] that includes the 43-item and newer 30-item MEQ (MEQ43 [56] and MEQ30 [57]). The German version of the 49-item Addiction Research Center Inventory (ARCI) [58, 59] was administered 11 h after drug administration. The duration of acute subjective effects was assessed using VAS “any drug effect” effect–time plots and an on/off threshold of 10% of the maximum individual response in Phoenix WinNonlin 6.4. Participants with responses <10% on this scale were not used to determine the effect duration (0, 3, and 4 participants for LSD, MDMA, and d-amphetamine, respectively).

#### Autonomic effects and adverse effects

Blood pressure, heart rate, and tympanic body temperature were repeatedly measured 1 and 0.5 h before and 0, 0.5, 1, 1.5, 2.5, 3, 4, 5, 6, 7, 8, 9, 10, and 11 h after drug administration (time specifications correspond to MDMA administration) as previously described in detail [60]. Pupil function was measured under standardized dark-light conditions and assessed using a Voltcraft MS-1300 luxmeter (Voltcraft, Hirschau, Germany) after a dark adaption time of 1 min as previously described [61]. Adverse effects were assessed 1 h before and 11 h after drug administration using the 66-item List of Complaints [62]. This scale yields a total adverse effects score and reliably measures physical and general discomfort.

#### Endocrine effects

Plasma levels of oxytocin were measured at baseline and 1.5, 2.5, 3, and 5 h after MDMA administration. Oxytocin concentrations were measured using the oxytocin enzyme-linked immunosorbent assay (ELISA) kit (ENZO Life Sciences, Ann Arbor, MI) according to the manufacturer’s protocol as previously described [63]. The plasma levels of BDNF were measured at baseline and 3 and 5 h after drug administration. Plasma BDNF levels were measured using an ELISA kit (Biosensis Mature BDNF Rapid ELISA kit: human, mouse, rat; Thebarton, Australia) as previously described [64]. Analyses were performed at the end of the study in one batch.

### Plasma drug concentrations

The plasma levels of LSD, d-amphetamine, and the LSD metabolite O-H-LSD were measured at baseline and 1, 1.5, 2, 3, 3.5, 4.5, 5.5, 6.5, 7.5, 9.5, and 11.5 h after drug administration. The plasma levels of MDMA and MDMA metabolites 3,4-methylenedioxyamphetamine (MDA) and 4-hydroxy-3-methoxymethamphetamine (HMMA) were measured at baseline and 0, 0.5, 1, 1.5, 2.5, 3, 4, 5, 6, 7, 9, and 11 h after drug administration using liquid chromatography-tandem mass spectrometry as previously described [28, 32, 50]. The data were analyzed using non-compartmental analysis.

### Statistical analyses

For measures repeatedly taken over time during each session, we first determined the peak effects (*E*_max_ and/or *E*_min_) or peak changes from baseline (Table 1). The values were then analyzed using repeated-measures analysis of variance, with drug as the sole within-subjects factor, followed by Tukey’s post hoc comparisons based on significant main effects. The criterion for significance was *p* < 0.05.

**Table 1:** Comparison of the acute effects of LSD, MDMA, d-amphetamine, and placebo

		Placebo (mean ± SEM)	LSD (mean ± SEM)	MDMA (mean ± SEM)	d-Amphetamine (mean ± SEM)	*F*_3,78_	*P*
Subjective effects							
VAS (%max)							
Any drug effect	Δ*E*_max_	1.6 ± 1.0	87 ± 3.3***	59 ± 5.8***^,###^	37 ± 4.8***^,###,†††^	114.94	<0.001
Good drug effect	Δ*E*_max_	3.0 ± 2.5	82 ± 3.6***	64 ± 5.9***^,##^	45 ± 4.8***^,###,††^	89.09	<0.001
Bad drug effect	Δ*E*_max_	0.1 ± 0.1	31 ± 5.3***	8.7 ± 3.1^###^	4.9 ± 1.9^###^	18.26	<0.001
Drug liking	Δ*E*_max_	2.8 ± 2.4	76 ± 4.4***	64 ± 6.1***	48 ± 5.0***^,###,†^	63.95	<0.001
Drug high	Δ*E*_max_	3.7 ± 2.8	70 ± 5.9***	58 ± 6.5***	41 ± 6.0***^,###,†^	40.81	<0.001
Stimulated	Δ*E*_max_	3.4 ± 2.6	69 ± 6.1***	56 ± 6.6***	46 ± 6.0***^,##^	40.77	<0.001
Ego dissolution	Δ*E*_max_	0.9 ± 0.7	83 ± 10.2***	44 ± 7.9**^,###^	50 ± 13.0^###,†^	60.95	<0.001
Open	Δ*E*_max_	1.5 ± 1.0	21 ± 3.7***	24 ± 3.4***	22 ± 3.3***	13.02	<0.001
Talkative	Δ*E*_max_	1.2 ± 1.0	17 ± 3.2***	20 ± 3.5***	24 ± 3.0***	16.32	<0.001
	Δ*E*_min_	−0.5 ± 0.5	−31 ± 3.5***	−12 ± 3.1*^,###^	−4.7 ± 2.0^###^	32.05	<0.001
Concentration	Δ*E*_max_	0.0 ± 0.0	6.6 ± 2.4	11 ± 3.0**	15 ± 2.8***	7.90	<0.001
	Δ*E*_min_	−0.8 ± 0.6	−38 ± 2.6***	−20 ± 3.3***^,###^	−5.3 ± 1.3^###,†††^	65.97	<0.001
Sense of time	Δ*E*_max_	0.0 ± 0.0	10 ± 3.3**	6.7 ± 2.3	3.3 ± 1.2	4.81	<0.01
	Δ*E*_min_	−1.3 ± 1.1	−40 ± 2.5***	−12 ± 3.0**^,###^	−1.6 ± 0.6^###,†^	79.92	<0.001
Speed of thinking	Δ*E*_max_	0.0 ± 0.0	11 ± 3.4**	9.4 ± 2.9	8.6 ± 2.0	4.16	<0.01
	Δ*E*_min_	0.0 ± 0.0	−33 ± 2.9***	−15 ± 3.2***^,###^	−2.3 ± 0.7^###,††^	47.91	<0.001
AMRS score							
Activity	Δ*E*_max_	0.3 ± 0.3	−0.1 ± 0.3	0.9 ± 0.4	2.2 ± 0.5**^,###^	6.74	<0.001
Concentration	Δ*E*_max_	−0.1 ± 0.3	−0.7 ± 0.7	0.2 ± 0.4	1.5 ± 0.4^##^	4.05	<0.01
Extroversion	Δ*E*_max_	0.2 ± 0.3	−0.1 ± 0.5	2.4 ± 0.4**^,###^	2.7 ± 0.5***^,###^	11.70	<0.001
Introversion	Δ*E*_max_	0.1 ± 0.1	5.7 ± 0.6***	2.1 ± 0.4**^,###^	0.8 ± 0.2###	45.37	<0.001
Inactivity	Δ*E*_max_	0.4 ± 0.2	4.1 ± 0.6***	1.8 ± 0.4^###^	0.7 ± 0.2^###^	19.47	<0.001
Well-being	Δ*E*_max_	0.4 ± 0.4	2.9 ± 1.1	4.7 ± 0.7***	4.8 ± 0.8***	7.49	<0.001
Emotional excitation	Δ*E*_max_	−0.5 ± 0.3	4.8 ± 1.2***	1.9 ± 0.5^##^	1.9 ± 0.6*^,#^	11.66	<0.001
Anxiety	Δ*E*_max_	−0.2 ± 0.1	1.3 ± 0.5***	0.3 ± 0.2^#^	0.1 ± 0.1^##^	6.76	<0.001
Autonomic effects							
Systolic blood pressure (mmHg)	*E*_max_	129 ± 2.1	140 ± 2.6***	149 ± 2.8***^,##^	161 ± 2.9***^,###,†††^	70.33	<0.001
Diastolic blood pressure (mmHg)	*E*_max_	79 ± 1.3	88 ± 1.4***	89 ± 1.4***	97 ± 1.7***^,###,†††^	61.42	<0.001
Heart rate (beats/min)	*E*_max_	77 ± 2.0	92 ± 3.0***	88 ± 2.3***	87 ± 3.0***	20.20	<0.001
Rate–pressure product (beats∙mmHg/min)	*E*_max_	9639 ± 329	12725 ± 578***	12707 ± 440***	12042 ± 484***	32.14	<0.001
Body temperature (°C)	*E*_max_	37.2 ± 0.1	37.6 ± 0.1***	37.5 ± 0.0***	37.6 ± 0.1***^,†^	22.76	<0.001
Pupil size (mm)	*E*_max_	6.3 ± 0.1	7.0 ± 0.1***	7.1 ± 0.1***	7.1 ± 0.1***	59.23	<0.001
Pupil size after light stimulus (mm)	*E*_max_	4.4 ± 0.1	5.4 ± 0.1***	6.3 ± 0.2***^,###^	5.4 ± 0.1***^,†††^	110.51	<0.001
Constriction amplitude (mm)	*E*_min_	1.7 ± 0.1	1.4 ± 0.1*	0.8 ± 0.1***^,###^	1.61 ± 0.04^†††^	49.36	<0.001
LC score							
Acute adverse effects	0–11 h	2.9 ± 1.3	8.15 ± 2.02*	5.43 ± 1.0	5.64 ± 1.37	2.63	NS
Hormones							
BDNF (mU/L)	*E*_max_	2974 ± 425	2524 ± 370	3001 ± 423	2153 ± 265	1.27	NS
Oxytocin (pg/mL)	*E*_max_	259 ± 62	279 ± 60	809 ± 64***^,###^	194 ± 35^†††^	27.36	<0.001

*VAS* visual analoge scale, *AMRS* Adjective Mood Rating Scale, *LS* List of Complaints, *NS* not significant, *E*_max_ maximal effect, *ΔE*_max_ maximal difference from baseline

*P < 0.05, **P < 0.01, ***P < 0.001 compared with placebo; ^#^*P* < 0.05, ^##^*P* < 0.01, ^###^*P* < 0.001 compared with LSD; ^†^*P* < 0.05, ^††^*P* < 0.01, ^†††^*P* < 0.001 compared with MDMA

## Results

All 28 participants completed the MDMA, d-amphetamine, and placebo session. One participant quit before the final LSD session and only the data from the other sessions was included in the analysis.

### Subjective mood effects

**Fig. 1:**
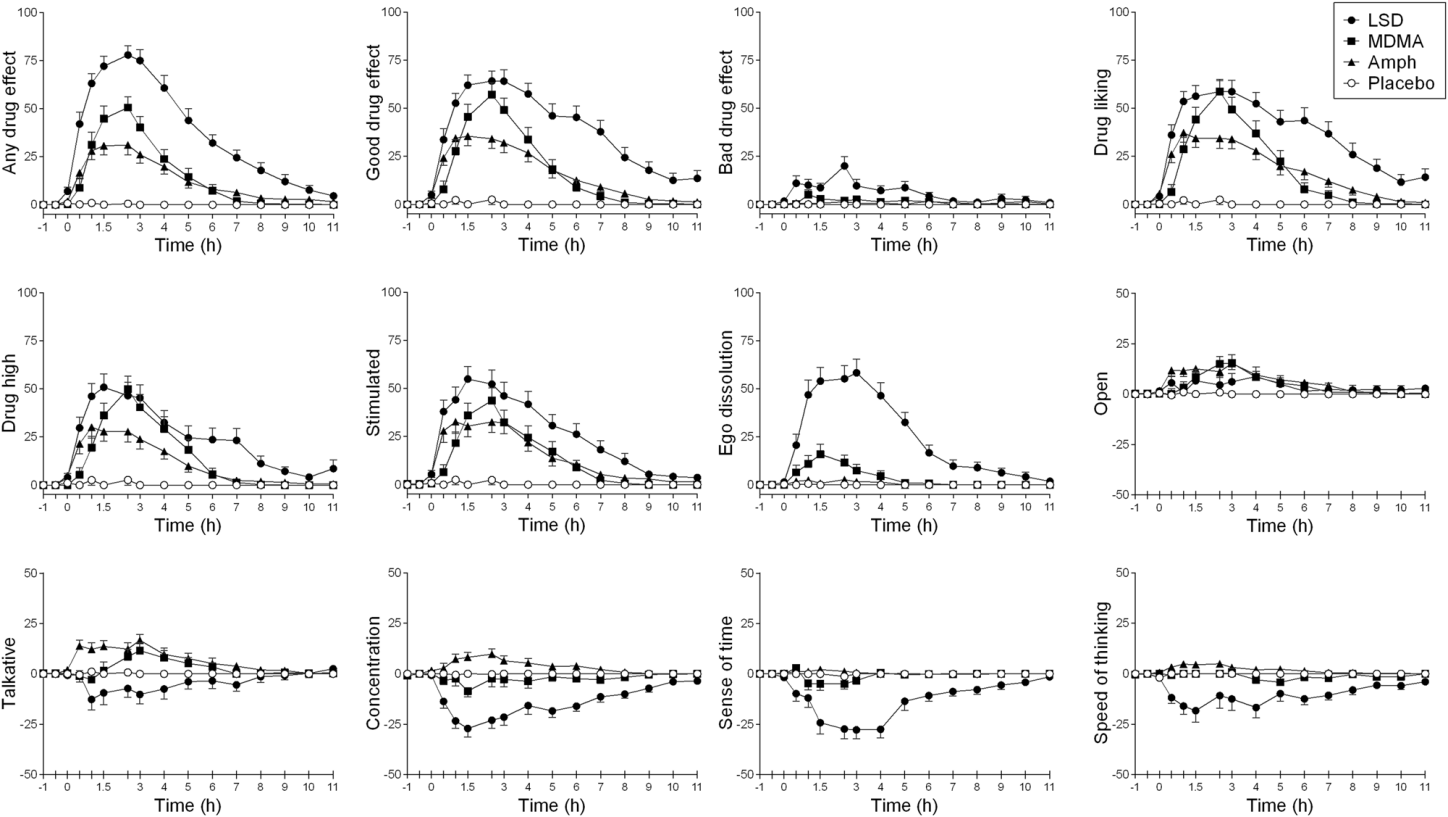
Subjective effects of LSD, MDMA, and d-amphetamine over time on the VASs. The data are expressed as mean ± SEM. LSD produced significantly greater ratings of “any drug effect,” “good drug effect,” “bad drug effect,” and “ego dissolution” compared with MDMA and d-amphetamine. In contrast, LSD reduced ratings of “talkative,” “concentration,” “sense of time,” and “speed of thinking” compared with MDMA and d-amphetamine. MDMA produced greater ratings of “any drug effect,” “good drug effect,” “liking,” “high,” and “ego dissolution” compared with d-amphetamine. The corresponding maximal responses and statistics are shown in Table 1.

Subjective effects were measured over time using VASs (Fig. 1). The corresponding peak responses are presented in Table 1. LSD produced an overall greater response than both MDMA and d-amphetamine, reflected by significantly higher increases in ratings of “any drug effect,” “good drug effect,” “bad drug effect,” and “ego dissolution” compared with MDMA and d-amphetamine. LSD also produced greater “drug liking,” “drug high,” and “stimulation” than d-amphetamine, whereas the effects of LSD on these scales did not significantly differ from MDMA. MDMA and d-amphetamine but not LSD increased peak ratings of “concentration” compared with placebo and LSD (Table 1). In contrast, LSD induced greater mean reductions over time (Fig. 1) and greater maximal reductions of ratings of talkative, concentration, sense of time, and speed of thinking compared with MDMA and d-amphetamine (Table 1). Only LSD and not MDMA or d-amphetamine induced significant “bad drug effects” compared with placebo. The overall effects (“any drug effect”) of LSD, MDMA, and d-amphetamine lasted (mean ± SD) 8.5 ± 2.0 h, 4.4 ± 1.7 h, and 6.2 ± 2.0 h, respectively.

All three drugs similarly increased ratings of feeling “talkative” and “open.” MDMA produced higher ratings of “any drug effect,” “good drug effect,” “drug liking,” and “drug high” compared with d-amphetamine.

**Fig. 2:**
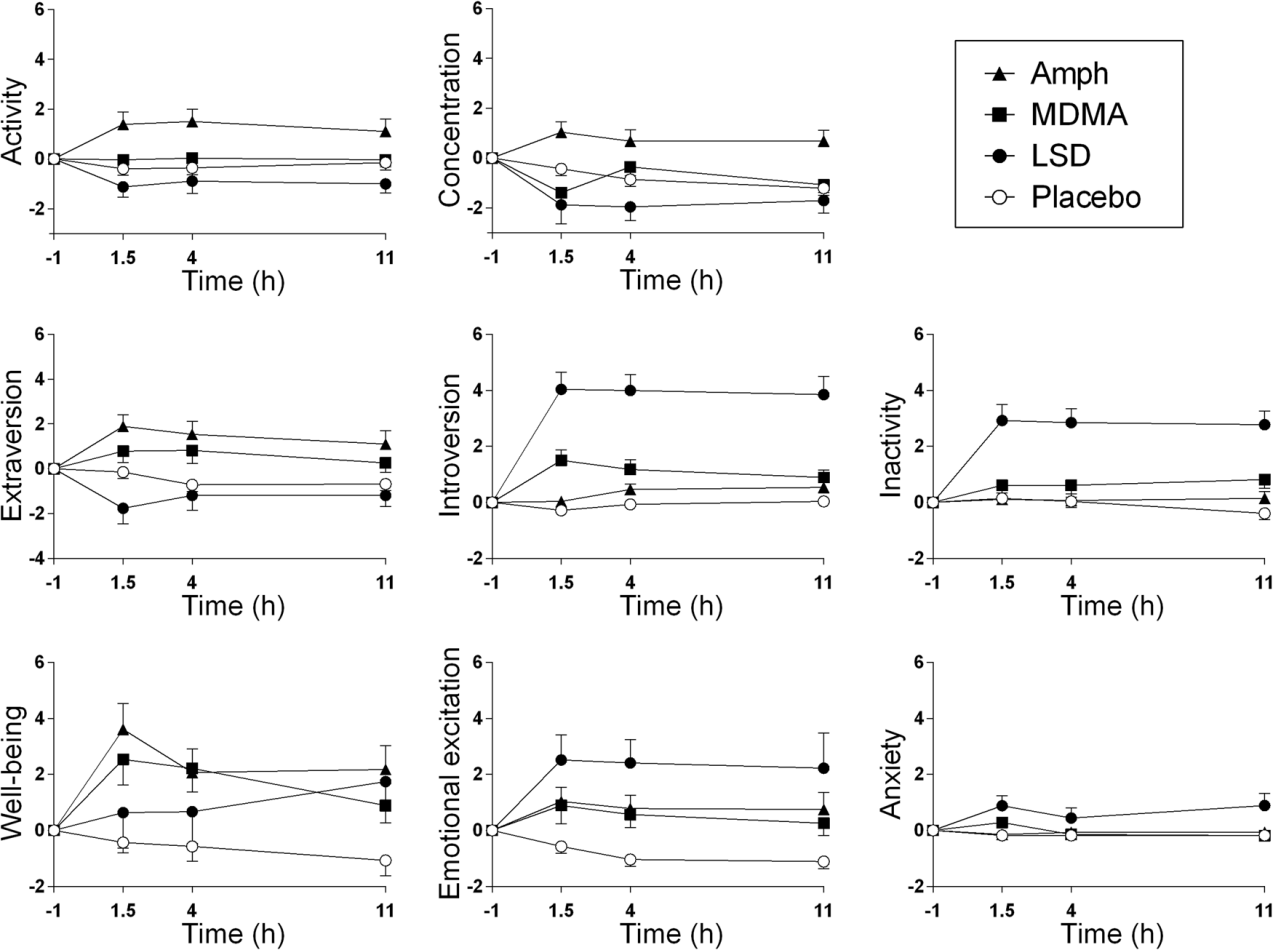
Subjective effects of LSD, MDMA, and d-amphetamine over time on the AMRS. The data are expressed as mean ± SEM changes from baseline. d-Amphetamine increased ratings of activity and concentration compared with LSD. LSD increased ratings of inactivity compared with MDMA and d-amphetamine. LSD increased introversion and reduced extraversion compared with MDMA and d-amphetamine. MDMA and d-amphetamine increased ratings of well-being compared with placebo, whereas LSD produced no significant effect compared with placebo, and its effects did not differ from MDMA or d-amphetamine. LSD significantly increased emotional excitation and anxiety compared with MDMA and D-amphetamine. The corresponding maximal effects and statistics are shown in Table 1.

On the AMRS (Fig. 2, Table 1), LSD produced greater “introversion,” “inactivity,” “emotional excitation,” and “anxiety” compared with MDMA and d-amphetamine. Conversely, MDMA and d-amphetamine increased “extraversion” compared with LSD. d-Amphetamine also increased “activity” and “concentration” compared with LSD.

**Fig. 3:**
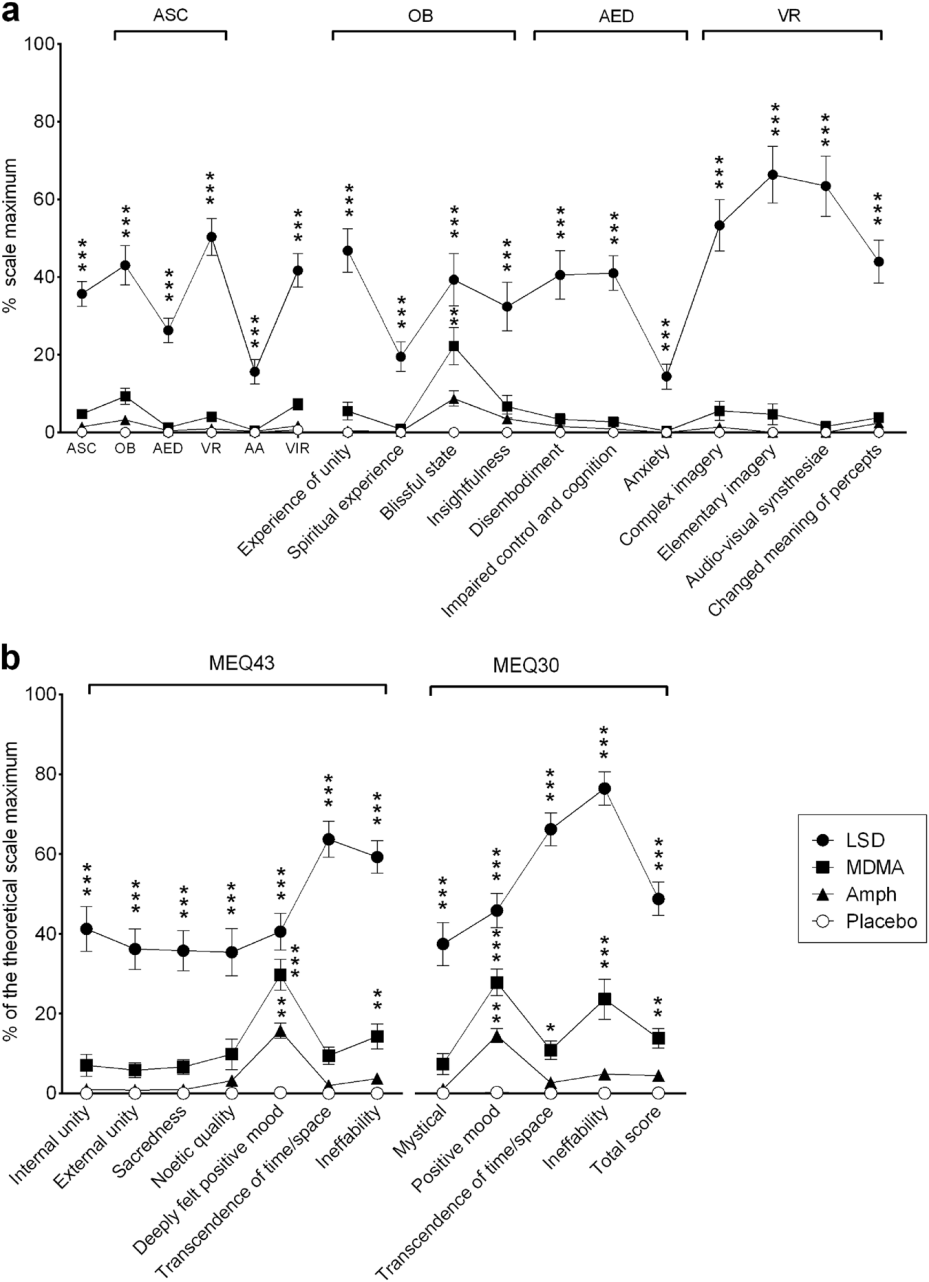
Subjective effects of LSD, MDMA, and d-amphetamine on the 5D-ASC scale and MEQ. The data are expressed as mean ± SEM. *p < 0.05, **p < 0.01, ***p < 0.001, vs. placebo. **a** LSD produced significantly greater ratings on all dimensions and subscales of the 5D-ASC scale compared with MDMA, d-amphetamine, and placebo. The effects of MDMA tended to be greater than d-amphetamine, but these differences were not statistically significant. MDMA produced significant increases only on the blissful state subscale compared with placebo. The effects of d-amphetamine did not differ significantly from placebo on any of the scales. The corresponding maximal effects and statistics are shown in Table S1. **b** LSD produced significantly higher ratings on all scales of the MEQ43 and MEQ30 compared with MDMA, d-amphetamine, and placebo, with the exception of nonsignificantly different positive mood ratings for LSD and MDMA on the MEQ43. MDMA significantly increased positive mood and ineffability ratings on the MEQ43 and MEQ30 compared with placebo. d-Amphetamine significantly increased positive mood ratings on the MEQ43 and MEQ30, but these effects were significantly lower than MDMA. The corresponding maximal effects and statistics are shown in Table S1.

LSD was the only drug that induced marked alterations of mind, reflected by large increases on all subscales of the 5D-ASC (Fig. 3a, Supplementary Table S1) compared with placebo, MDMA (Tukey’s post hoc tests: *p* < 0.001 for all comparisons), and d-amphetamine (*p* < 0.001 for all comparisons). MDMA only significantly increased ratings of “blissful state” compared with placebo, whereas d-amphetamine had no significant effects on any of the 5D-ASC subscales.

LSD increased ratings on all scales of the MEQ43 and MEQ30 compared with MDMA, d-amphetamine, and placebo (*p* < 0.001 for all comparisons), with the exception of nonsignificant differences in ratings of “deeply felt positive mood” for LSD and MDMA on the MEQ43 (Fig. 3b, Supplementary Table S1). MDMA significantly increased ratings of positive mood and ineffability (difficulty describing the experience in words) on the MEQ43 and MEQ30 compared with placebo (*p* < 0.01). d-Amphetamine moderately increased positive mood ratings on the MEQ43 and MEQ30.

On the ARCI, LSD increased ratings on all subscales that indicated broad (mixed) hallucinogenic, sedative, and euphoriant effects (Supplementary Fig. S1), with the exception of a decrease on the benzedrine group scale, indicating lower stimulation. In contrast, d-amphetamine was the only drug that increased ratings on the benzedrine group scale.

### Vital signs and adverse effects

**Fig. 4:**
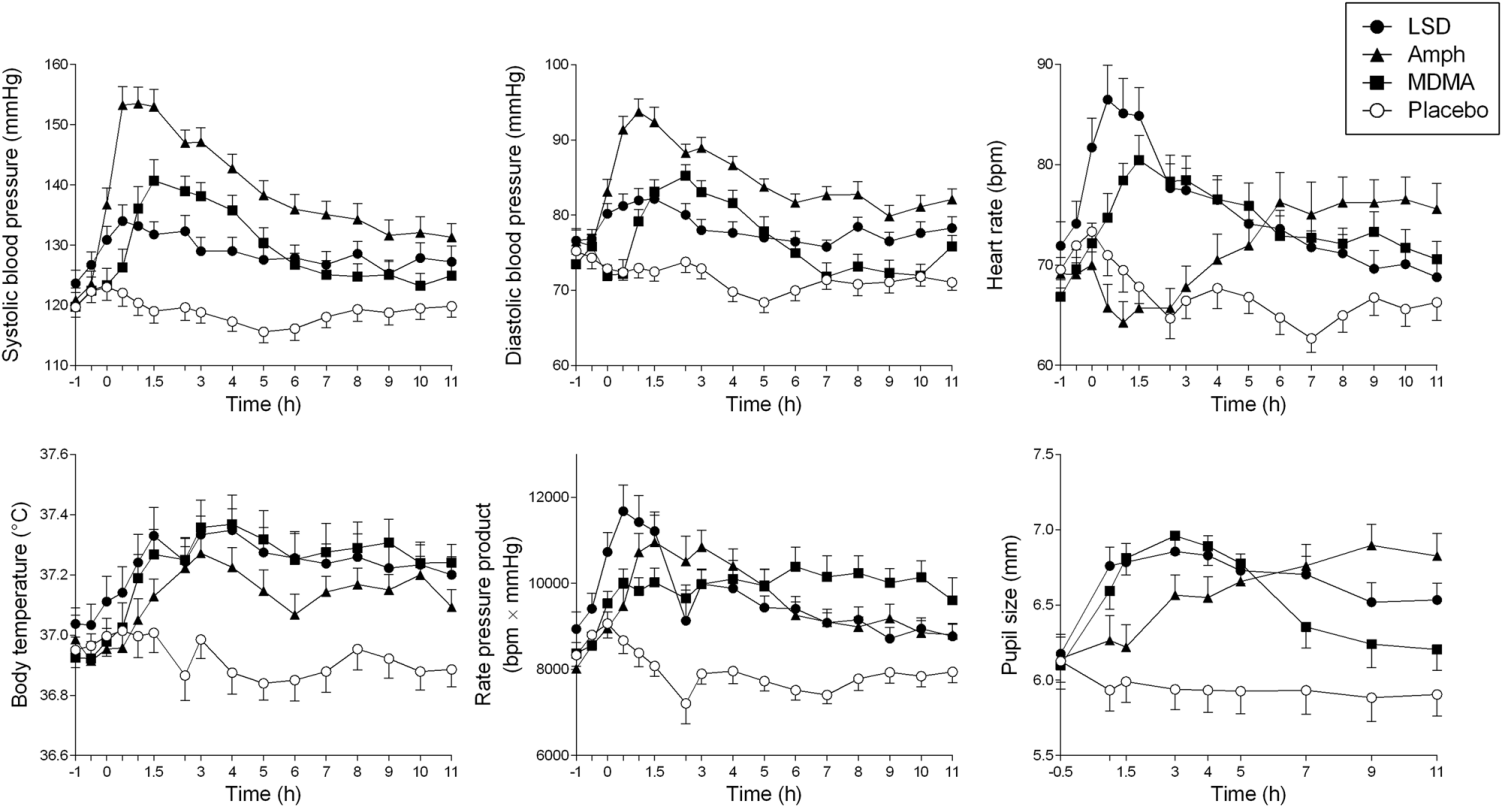
Autonomic responses to LSD, MDMA, d-amphetamine, and placebo. The data are expressed as mean ± SEM. All of the active substances produced significant sympathomimetic stimulation, reflected by increases in systolic and diastolic blood pressure, heart rate, body temperature, and pupil size. Importantly, the overall hemodynamic response, expressed as the rate–pressure product, was similarly increased by all of the active substances compared with placebo. However, d-amphetamine produced significantly higher increases in blood pressure than LSD and MDMA. Conversely, LSD and MDMA produced greater increases in heart rate than d-amphetamine during the first 4 h. The corresponding maximal effects and statistics are shown in Table 1.

The effects of the drugs on vital signs over time are shown in Fig. 4, and peak effects are shown in Table 1. All active substances significantly increased blood pressure, heart rate, and body temperature compared with placebo. Systolic hypertension > 140 mmHg was seen in 23, 18, 14, and 3 participants after d-amphetamine, MDMA, LSD, and placebo, respectively. Tachykardia >100 beats/min was seen in 5, 5, 7, and 0 participants after d-amphetamine, MDMA, LSD, and placebo, respectively. d-Amphetamine produced a significantly higher increase in blood pressure compared with LSD and MDMA, and LSD and MDMA produced lower heart rate increases than d-amphetamine over the first 4 h, but all three drugs produced overall similar hemodynamic stimulation, considering the similar increases in the rate–pressure product. All three substances increased pupil size (Fig. 4, Table 1). However, only MDMA markedly and significantly impaired normal light-induced pupil constriction compared with placebo (Table 1, Supplementary Fig. S2). Only LSD increased the total acute (0–11 h) adverse effects score on the List of Complaints compared with placebo. Frequently reported adverse effects are presented in Supplementary Table S2. No severe adverse events were observed.

### Endocrine effects

MDMA but not LSD or d-amphetamine increased plasma concentrations of oxytocin (Fig. S4, Table 1). None of the substances altered plasma concentrations of BDNF (Fig. S4, Table 1).

### Plasma drug concentrations

The concentration–time curves for LSD, O-H-LSD, d-amphetamine, MDMA, MDA, and HMMA are shown in Supplementary Fig. S3. The geometric mean maximum (*C*_max_) values (range) for LSD and O-H-LSD were 1.8 (0.99–2.9) and 0.12 (0.07–0.2) ng/ml, respectively. The *T*_max_ values were 1.6 (1–3.5) and 5.2 (3.1–7.5) h, respectively. The *C*_max_ values for MDMA, MDA, and HMMA were 236 (158–357), 10.9 (5.3–19), and 160 (43–287) ng/ml, respectively. The corresponding *T*_max_ values were 3.0 (1.1–5.0), 7.0 (3.0–11), and 2.8 (1.3–6.0) h, respectively. The *C*_max_ and *T*_max_ values for d-amphetamine were 100 (68–133) ng/ml and 2.6 (1.0–5.5) h, respectively.

### Blinding

Data on the participants’ retrospective identification of the study substances are shown in Supplementary Table S3. All of the participants correctly identified placebo, 96% correctly identified LSD, 75% correctly identified MDMA, and 75% correctly identified d-amphetamine. MDMA was misclassified as d-amphetamine and vice versa (21%). One participant (4%) misidentified LSD as MDMA and vice versa. One participant (4%) identified d-amphetamine as placebo. Thus, LSD was well distinguished from MDMA and d-amphetamine.

## Discussion

As hypothesized, LSD produced stronger and more distinct subjective effects compared with MDMA and d-amphetamine. Specifically, only LSD induced significant and marked alterations of consciousness on all 5D-ASC and MEQ subscales compared with placebo, and responses were also significantly greater compared with MDMA and d-amphetamine. In contrast, MDMA only moderately increased “blissful state” on the 5D-ASC scale and “positive mood” and “ineffability” on the MEQ. d-Amphetamine only weakly increased “positive mood” on the MEQ compared with placebo. Additionally, LSD produced greater overall subjective effects, including both “good drug effects” and “bad drug effects,” on the VAS compared with both MDMA and d-amphetamine. Only LSD produced significant “bad drug effects” on the VAS, “anxiety” on the 5D-ASC scale, and “LSD group” effects and “pentobarbital-chlorpromazine-alcohol group” effects on the ARCI compared with placebo. Finally, LSD was correctly identified by 96% and 100% of the participants on the day of administration and at the end of the study, respectively. However, similarities were also observed in the effects of all compounds on scales that measured positive drug effects. All of the drugs produced comparable ratings of “open” and “talkative” on the VAS, and ratings of “drug high,” “drug liking,” and “stimulated” on the VAS did not differ between LSD and MDMA. The present findings are overall consistent with previous reports on the effects of LSD [3, 4, 38, 50, 65], MDMA [18, 25, 28], and d-amphetamine [32]. In contrast to these previous studies, however, the present study compared the subjective responses to LSD, MDMA, and d-amphetamine using a within-subjects design. Subjective effects of various substances can differ, depending on the comparator that is used. For example, marked effects of MDMA on the 5D-ASC scale compared with inactive placebo have been previously reported [18]. However, when MDMA was compared with LSD in the present study, it induced only minimal and comparatively weak alterations of consciousness.

The present findings have clinical implications. First, acute effects of the LSD-like hallucinogen psilocybin on both the 5D-ASC scale and MEQ also used in the present study have been shown to predict long-term therapeutic outcomes in patients with anxiety and depression in previous studies [6–8]. Similarly, 5D-ASC scale and MEQ ratings correlated with changes in well-being and life satisfaction 1 year after LSD administration in healthy subjects in a previous study [10]. Thus, stronger acute responses to LSD on the 5D-ASC scale and MEQ, as documented in the present study in healthy participants and previously in patients [37], may also predict better therapeutic outcomes in studies that evaluate the benefits of LSD-assisted psychotherapy in patients with anxiety and depression [66, 67]. However, this assumption needs to be verified in patients. Second, the present study found that MDMA produced some qualitatively similar (although less pronounced) positive effects compared with LSD, but with lower associated “bad drug effects” and anxiety. Thus, MDMA may produce less untoward effects than LSD, and this may favor its use in patients afraid to take LSD or at risk of adverse reaction (i.e., high neuroticism, high emotional lability, and young age [47]). In fact, MDMA is often used prior to LSD in substance-assisted psychotherapy in Switzerland so that patients can familiarize themselves with substance-induced states [66, 68, 69]. For example, MDMA could be used prior to LSD or psilocybin in substance-assisted psychotherapy so that patients can familiarize themselves with substance-induced states. In fact, MDMA has often been used in the first 1–3 sessions before the use of LSD in substance-assisted psychotherapy in Switzerland.

In the present study, we also directly compared the acute effects of MDMA and d-amphetamine and we hypothesized that MDMA would produce distinct subjective emotional effects compared with d-amphetamine. Previous studies have discussed the extent to which the effects of these amphetamines differ [25, 27, 28, 70]. The present study supports the view that the empathogen MDMA produces at least some clearly distinct effects compared with a pure stimulant, such as d-amphetamine. In the present study, MDMA produced greater ratings of “any drug effect,” “good drug effect,” “drug high,” and “drug liking” on the VAS, greater ratings of “positive mood” on the MEQ, and smaller “benzedrine group” effects on the ARCI than d-amphetamine. MDMA also induced greater impairments in “concentration” and “speed of thinking” compared with d-amphetamine.

In contrast and as predicted, MDMA but not d-amphetamine increased plasma oxytocin concentrations, which is thought to be attributable to the MDMA-induced release of 5-HT and 5-HT_1A_ receptor stimulation [23]. Interestingly, the potent 5-HT_1A_ and 5-HT_2A_ receptor agonist LSD [5] did not significantly increase plasma oxytocin levels in the present study, in contrast to a higher dose of LSD and inactive placebo as the comparator in a previous study [3]. Supporting the view of distinct effects of MDMA and d-amphetamine, 75% and 89% of the participants in the present study correctly identified MDMA and d-amphetamine on the day of administration and at the end of the study, respectively. However, MDMA and d-amphetamine also produced overlapping effects, including comparable increases in “open” and “talkative” on the VAS, “well-being” and “extraversion” on the AMRS, and a lack of significant “bad drug effects” or “anxiety” compared with placebo and in contrast to LSD. Similar partly overlapping effects of MDMA and lower doses of d-amphetamine (10–20 mg) have been previously reported [33, 71]. Interestingly, both MDMA and d-amphetamine seemed to produce relatively comparable “empathogenic” effects in the present study, whereas such effects were somewhat more unique to MDMA compared with the stimulant methylphenidate [27, 28]. Thus, MDMA and d-amphetamine are more alike than MDMA and methylphenidate, but this remains to be clarified in future studies. Pharmacologically, d-amphetamine and methylphenidate both activate the dopamine and norepinephrine systems without having relevant effects on 5-HT. However, d-amphetamine also releases monoamines similarly to MDMA, in contrast to the pure uptake inhibitor methylphenidate [29, 72].

In the present study, LSD, MDMA, and d-amphetamine produced comparable sympathomimetic activation, reflected by similar increases in the rate–pressure product, body temperature, and pupil size. Additionally, LSD, MDMA, and d-amphetamine produced comparable amounts of total adverse effects as evidenced by similar scores on the List of Complaints (Table 1), although there were some differences between the substances regarding the specific complaints (Table S2). These findings indicate that the doses of the drugs were similar with regard to sympathomimetic effects, including cardiovascular system stimulation and somatic complaints. The finding that LSD produced relatively pronounced sympathomimetic effects confirmed our previous studies [3, 38] and contradicted the assumption that LSD does not increase blood pressure [67]. On the other hand, the study findings suggest that LSD is capable of inducing greater acute psychological effects (positive and negative) than MDMA and d-amphetamine at doses that are producing comparable somatic adverse responses.

In the present study, we also determined plasma drug concentrations. Peak concentrations of MDMA and d-amphetamine were similar to previous studies that tested identical doses [32, 39, 73]. The full pharmacokinetic data for LSD derived from the present study have been published elsewhere [50]. Importantly, slightly higher plasma concentrations of LSD were documented in the present study compared with a previous study that reportedly used the same dose (0.1 mg) [49]. The higher plasma concentrations in the present study can be explained by the use of a higher dose (0.096 mg) of LSD base (analytically confirmed content and stability) compared with a lower estimated dose of 0.070 mg in previous studies [38, 49], as discussed previously [50].

The main strength and novelty of the present study was that we employed a double-blind, placebo-controlled, within-subjects design that included different active substances and validated pharmacodynamic and substance concentration measurements. The present study also has limitations. We only used one dose level of each substance. Full dose–response curves would need to be generated for each substance to achieve valid comparisons. However, we used a relatively low dose of LSD compared with the doses of MDMA and d-amphetamine and nevertheless found stronger effects of LSD compared with MDMA and d-amphetamine. Additionally, a previous study that used a higher dose of LSD (0.2 mg) showed significantly greater acute subjective effects of LSD compared with 0.1 mg LSD (the dose used in the present study), but autonomic stimulation was similar between doses [38]. Specifically, the higher dose produced both greater “good drug effect” and “bad drug effect” ratings on the VASs [38] and higher ratings of “blissful state,” “insightfulness,” and “changed meaning of percepts,” but no increase in “anxiety” on the 5D-ASC [37] compared with the lower dose of LSD. Thus, both desired and untoward drug effects were dose-dependent and future multiple dose-level studies will be needed to further define ideal dose ranges. Thus, higher doses of LSD up to 0.2 mg that are already clinically used [2, 67] can be expected to produce even greater subjective effects than the dose (0.1 mg) that was used in the present study. The dose of MDMA that was used in the present study is in the upper range of doses that are used clinically; higher doses would not likely produce stronger positive subjective effects, but would likely result in more adverse somatic responses [39]. Finally, we found that the doses of all of the active substances were equivalent with regard to autonomic stimulation. Nevertheless, there is a need for additional studies including multiple dose levels and additional outcomes such as imaging.

In conclusion, the present study found that LSD induced different and more pronounced alterations of waking consciousness compared with MDMA and d-amphetamine in the same subjects. MDMA also showed partly distinct effects compared with d-amphetamine. The acute-effect profiles of LSD and MDMA will be useful to assist the dose selection for substance-assisted psychotherapy research and to inform patients and researchers on what to expect in terms of positive and negative acute responses to these substances.

## Funding and disclosure

The authors declare no competing financial interests. This work was supported by the Swiss National Science Foundation (grant no. 320030_170249).

## Supplementary information

Supplemental File - Consort Flowchart

Supplementary Data

Figure S1

Figure S2

Figure S3

Figure S4

Table S1

Table S2

Table S3

## References

[1] Krebs TS, Johansen PO (2013). Over 30 million psychedelic users in the United States. F1000 Res.

[2] Liechti ME (2017). Modern clinical research on LSD. Neuropsychopharmacology.

[3] Schmid Y, Enzler F, Gasser P, Grouzmann E, Preller KH, Vollenweider FX (2015). Acute effects of lysergic acid diethylamide in healthy subjects. Biol Psychiatry.

[4] Preller KH, Herdener M, Pokorny T, Planzer A, Kraehenmann R, Stämpfli P (2017). The fabric of meaning and subjective effects in LSD-induced states depend on serotonin 2A receptor activation. Curr Biol.

[5] Rickli A, Moning OD, Hoener MC, Liechti ME (2016). Receptor interaction profiles of novel psychoactive tryptamines compared with classic hallucinogens. Eur Neuropsychopharmacol.

[6] Roseman L, Nutt DJ, Carhart-Harris RL (2017). Quality of acute psychedelic experience predicts therapeutic efficacy of psilocybin for treatment-resistant depression. Front Pharmacol.

[7] Griffiths RR, Johnson MW, Carducci MA, Umbricht A, Richards WA, Richards BD (2016). Psilocybin produces substantial and sustained decreases in depression and anxiety in patients with life-threatening cancer: a randomized double-blind trial. J Psychopharmacol.

[8] Ross S, Bossis A, Guss J, Agin-Liebes G, Malone T, Cohen B (2016). Rapid and sustained symptom reduction following psilocybin treatment for anxiety and depression in patients with life-threatening cancer: a randomized controlled trial. J Psychopharmacol.

[9] Griffiths R, Richards W, Johnson M, McCann U, Jesse R (2008). Mystical-type experiences occasioned by psilocybin mediate the attribution of personal meaning and spiritual significance 14 months later. J Psychopharmacol.

[10] Schmid Y, Liechti ME (2018). Long-lasting subjective effects of LSD in normal subjects. Psychopharmacology.

[11] Garcia-Romeu A, Griffiths RR, Johnson MW (2015). Psilocybin-occasioned mystical experiences in the treatment of tobacco addiction. Curr Drug Abus Rev.

[12] Mithoefer Michael C., Feduccia Allison A., Jerome Lisa, Mithoefer Anne, Wagner Mark, Walsh Zach, Hamilton Scott, Yazar-Klosinski Berra, Emerson Amy, Doblin Rick (2019). MDMA-assisted psychotherapy for treatment of PTSD: study design and rationale for phase 3 trials based on pooled analysis of six phase 2 randomized controlled trials. Psychopharmacology.

[13] Mithoefer MC, Mithoefer AT, Feduccia AA, Jerome L, Wagner M, Wymer J (2018). 3,4-Methylenedioxymethamphetamine (MDMA)-assisted psychotherapy for post-traumatic stress disorder in military veterans, firefighters, and police officers: a randomised, double-blind, dose–response, phase 2 clinical trial. Lancet Psychiatry.

[14] Hysek CM, Schmid Y, Simmler LD, Domes G, Heinrichs M, Eisenegger C (2014). MDMA enhances emotional empathy and prosocial behavior. Soc Cogn Affect Neurosci.

[15] Kirkpatrick MG, Lee R, Wardle MC, Jacob S, de Wit H (2014). Effects of MDMA and intranasal oxytocin on social and emotional processing. Neuropsychopharmacology.

[16] Hysek CM, Simmler LD, Nicola V, Vischer N, Donzelli M, Krähenbühl S (2012). Duloxetine inhibits effects of MDMA (“ecstasy”) in vitro and in humans in a randomized placebo-controlled laboratory study. PLoS ONE.

[17] Liechti ME, Baumann C, Gamma A, Vollenweider FX (2000). Acute psychological effects of 3,4-methylenedioxymethamphetamine (MDMA, “ecstasy”) are attenuated by the serotonin uptake inhibitor citalopram. Neuropsychopharmacology.

[18] Hysek CM, Simmler LD, Ineichen M, Grouzmann E, Hoener MC, Brenneisen R (2011). The norepinephrine transporter inhibitor reboxetine reduces stimulant effects of MDMA (“ecstasy”) in humans. Clin Pharm Ther.

[19] Liechti ME, Saur MR, Gamma A, Hell D, Vollenweider FX (2000). Psychological and physiological effects of MDMA (“ecstasy”) after pretreatment with the 5-HT_2_ antagonist ketanserin in healthy humans. Neuropsychopharmacology.

[20] Dumont GJ, Sweep FC, van der Steen R, Hermsen R, Donders AR, Touw DJ (2009). Increased oxytocin concentrations and prosocial feelings in humans after ecstasy (3,4-methylenedioxymethamphetamine) administration. Soc Neurosci.

[21] Francis SM, Kirkpatrick MG, de Wit H, Jacob S (2016). Urinary and plasma oxytocin changes in response to MDMA or intranasal oxytocin administration. Psychoneuroendocrinology.

[22] Ramos L, Hicks C, Kevin R, Caminer A, Narlawar R, Kassiou M (2013). Acute prosocial effects of oxytocin and vasopressin when given alone or in combination with 3,4-methylenedioxymethamphetamine in rats: involvement of the V_1A_ receptor. Neuropsychopharmacology.

[23] Thompson MR, Callaghan PD, Hunt GE, Cornish JL, McGregor IS (2007). A role for oxytocin and 5-HT_1A_ receptors in the prosocial effects of 3,4 methylenedioxymethamphetamine (“ecstasy”). Neuroscience.

[24] Nichols DE (1986). Differences between the mechanism of action of MDMA, MBDB, and the classic hallucinogens. Identification of a new therapeutic class: entactogens. J Psychoact Drugs.

[25] Bershad AK, Miller MA, Baggott MJ, de Wit H (2016). The effects of MDMA on socio-emotional processing: does MDMA differ from other stimulants?. J Psychopharmacol.

[26] Hysek CM, Simmler LD, Schillinger N, Meyer N, Schmid Y, Donzelli M (2014). Pharmacokinetic and pharmacodynamic effects of methylphenidate and MDMA administered alone and in combination. Int J Neuropsychopharmacol.

[27] Schmid Y, Hysek CM, Simmler LD, Crockett MJ, Quednow BB, Liechti ME (2014). Differential effects of MDMA and methylphenidate on social cognition. J Psychopharmacol.

[28] Dolder PC, Muller F, Schmid Y, Borgwardt SJ, Liechti ME (2018). Direct comparison of the acute subjective, emotional, autonomic, and endocrine effects of MDMA, methylphenidate, and modafinil in healthy subjects. Psychopharmacology.

[29] Simmler L, Buser T, Donzelli M, Schramm Y, Dieu LH, Huwyler J (2013). Pharmacological characterization of designer cathinones in vitro. Br J Pharm.

[30] Verrico CD, Miller GM, Madras BK (2007). MDMA (ecstasy) and human dopamine, norepinephrine, and serotonin transporters: implications for MDMA-induced neurotoxicity and treatment. Psychopharmacology.

[31] Dolder PC, Strajhar P, Vizeli P, Odermatt A, Liechti ME (2018). Acute effects of lisdexamfetamine and d-amphetamine on social cognition and cognitive performance in a placebo-controlled study in healthy subjects. Psychopharmacology.

[32] Dolder PC, Strajhar P, Vizeli P, Hammann F, Odermatt A, Liechti ME (2017). Pharmacokinetics and pharmacodynamics of lisdexamfetamine compared with D-amphetamine in healthy subjects. Front Pharm.

[33] Tancer M, Johanson CE (2003). Reinforcing, subjective, and physiological effects of MDMA in humans: a comparison with d-amphetamine and mCPP. Drug Alcohol depend.

[34] Newhouse PA, Belenky G, Thomas M, Thorne D, Sing HC, Fertig J (1989). The effects of d-amphetamine on arousal, cognition, and mood after prolonged total sleep deprivation. Neuropsychopharmacology.

[35] Rush CR, Essman WD, Simpson CA, Baker RW (2001). Reinforcing and subject-rated effects of methylphenidate and d-amphetamine in non-drug-abusing humans. J Clin Psychopharmacol.

[36] Hintzen A, Passie T (2010). The pharmacology of LSD: a critical review.

[37] Liechti ME, Dolder PC, Schmid Y (2017). Alterations in conciousness and mystical-type experiences after acute LSD in humans. Psychopharmacology.

[38] Dolder PC, Schmid Y, Mueller F, Borgwardt S, Liechti ME (2016). LSD acutely impairs fear recognition and enhances emotional empathy and sociality. Neuropsychopharmacology.

[39] Vizeli P, Liechti ME (2017). Safety pharmacology of acute MDMA administration in healthy subjects. J Psychopharmacol.

[40] Kirkpatrick MG, Baggott MJ, Mendelson JE, Galloway GP, Liechti ME, Hysek CM (2014). MDMA effects consistent across laboratories. Psychopharmacology.

[41] Brunt TM, Koeter MW, Niesink RJ, van den Brink W (2012). Linking the pharmacological content of ecstasy tablets to the subjective experiences of drug users. Psychopharmacology.

[42] Wardle MC, De Wit H (2012). Effects of amphetamine on reactivity to emotional stimuli. Psychopharmacology.

[43] de Wit H, Enggasser JL, Richards JB (2002). Acute administration of d-amphetamine decreases impulsivity in healthy volunteers. Neuropsychopharmacology.

[44] Weafer J, de Wit H (2013). Inattention, impulsive action, and subjective response to d-amphetamine. Drug Alcohol Depend.

[45] Haile CN, Murrough JW, Iosifescu DV, Chang LC, Al Jurdi RK, Foulkes A (2014). Plasma brain derived neurotrophic factor (BDNF) and response to ketamine in treatment-resistant depression. Int J Neuropsychopharmacol.

[46] Ly C, Greb AC, Cameron LP, Wong JM, Barragan EV, Wilson PC (2018). Psychedelics promote structural and functional neural plasticity. Cell Rep.

[47] Studerus E, Gamma A, Kometer M, Vollenweider FX (2012). Prediction of psilocybin response in healthy volunteers. PLoS ONE.

[48] Wong YN, King SP, Laughton WB, McCormick GC, Grebow PE (1998). Single-dose pharmacokinetics of modafinil and methylphenidate given alone or in combination in healthy male volunteers. J Clin Pharm.

[49] Dolder PC, Schmid Y, Steuer AE, Kraemer T, Rentsch KM, Hammann F (2017). Pharmacokinetics and pharmacodynamics of lysergic acid diethylamide in healthy subjects. Clin Pharmacokinet.

[50] Holze F, Duthaler U, Vizeli P, Muller F, Borgwardt S, Liechti ME (2019). Pharmacokinetics and subjective effects of a novel oral LSD formulation in healthy subjects. Br J Clin Pharm.

[51] Kirkpatrick M, Delton AW, Robertson TE, de Wit H (2015). Prosocial effects of MDMA: a measure of generosity. J Psychopharmacol.

[52] Kuypers KPC, Dolder PC, Ramaekers JG, Liechti ME (2017). Multifaceted empathy of healthy volunteers after single doses of MDMA: a pooled sample of placebo-controlled studies. J Psychopharmacol.

[53] Janke W, Debus G (1978). Die Eigenschaftswörterliste.

[54] Dittrich A (1998). The standardized psychometric assessment of altered states of consciousness (ASCs) in humans. Pharmacopsychiatry.

[55] Studerus E, Gamma A, Vollenweider FX (2010). Psychometric evaluation of the altered states of consciousness rating scale (OAV). PLoS ONE.

[56] Griffiths RR, Richards WA, McCann U, Jesse R (2006). Psilocybin can occasion mystical-type experiences having substantial and sustained personal meaning and spiritual significance. Psychopharmacology.

[57] Barrett FS, Johnson MW, Griffiths RR (2015). Validation of the revised Mystical Experience Questionnaire in experimental sessions with psilocybin. J Psychopharmacol.

[58] Martin WR, Sloan JW, Sapira JD, Jasinski DR (1971). Physiologic, subjective, and behavioral effects of amphetamine, methamphetamine, ephedrine, phenmetrazine, and methylphenidate in man. Clin Pharm Ther.

[59] Bopp G, Bender W, Schütz CG (2005). Validierung der Deutschen Version des Addiction Research Center Inventory (ARCI). Suchtmedizin.

[60] Hysek CM, Vollenweider FX, Liechti ME (2010). Effects of a β-blocker on the cardiovascular response to MDMA (ecstasy). Emerg Med J.

[61] Hysek CM, Liechti ME (2012). Effects of MDMA alone and after pretreatement with reboxetine, duloxetine, clonidine, carvedilol, and doxazosin on pupillary light reflex. Psychopharmacology.

[62] Zerssen DV (1976). Die Beschwerden-Liste. Münchener Informationssystem.

[63] Holt-Lunstad J, Birmingham WA, Light KC (2008). Influence of a “warm touch” support enhancement intervention among married couples on ambulatory blood pressure, oxytocin, alpha amylase, and cortisol. Psychosom Med.

[64] Akimoto H, Oshima S, Sugiyama T, Negishi A, Nemoto T, Kobayashi D (2019). Changes in brain metabolites related to stress resilience: Metabolomic analysis of the hippocampus in a rat model of depression. Behav Brain Res.

[65] Carhart-Harris RL, Kaelen M, Bolstridge M, Williams TM, Williams LT, Underwood R (2016). The paradoxical psychological effects of lysergic acid diethylamide (LSD). Psychol Med.

[66] Gasser P, Kirchner K, Passie T (2015). LSD-assisted psychotherapy for anxiety associated with a life-threatening disease: a qualitative study of acute and sustained subjective effects. J Psychopharmacol.

[67] Gasser P, Holstein D, Michel Y, Doblin R, Yazar-Klosinski B, Passie T (2014). Safety and efficacy of lysergic acid diethylamide-assisted psychotherapy for anxiety associated with life-threatening diseases. J Nerv Ment Dis.

[68] Gasser P (1996). Die psycholytische Therapie in der Schweiz von 1988-1993. Schweiz Arch Neurol Psychiatr.

[69] Oehen P, Traber R, Widmer V, Schnyder U (2013). A randomized, controlled pilot study of MDMA (±3,4-methylenedioxymethamphetamine)-assisted psychotherapy for treatment of resistant, chronic post-traumatic stress disorder (PTSD). J Psychopharmacol.

[70] Bedi G, Hyman D, de Wit H (2010). Is ecstasy an “empathogen”? Effects of ±3,4-methylenedioxymethamphetamine on prosocial feelings and identification of emotional states in others. Biol Psychiatry.

[71] Johanson CE, Kilbey M, Gatchalian K, Tancer M (2006). Discriminative stimulus effects of 3,4-methylenedioxymethamphetamine (MDMA) in humans trained to discriminate among d-amphetamine, *meta*-chlorophenylpiperazine and placebo. Drug Alcohol Depend.

[72] Simmler LD, Rickli A, Schramm Y, Hoener MC, Liechti ME (2014). Pharmacological profiles of aminoindanes, piperazines, and pipradrol derivatives. Biochem Pharmacol.

[73] Schmid Y, Vizeli P, Hysek CM, Prestin K, Meyer zu Schwabedissen HE, Liechti ME (2016). CYP2D6 function moderates the pharmacokinetics and pharmacodynamics of 3,4-methylene-dioxymethamphetamine in a controlled study in healthy subjects. Pharmacogenet Genom.

