# Supplementary material for: Distinct acute effects of LSD, MDMA, and d-amphetamine in healthy subjects
Source: Neuropsychopharmacology. 2019 Nov 16;45(3):462–71. doi: 10.1038/s41386-019-0569-3 (PMC6969135; doi:10.1038/s41386-019-0569-3)
Supplement: Supplementary file 1 — Supplemental File - Consort Flowchart [file 41386_2019_569_MOESM1_ESM.ppt]

## Slide 1
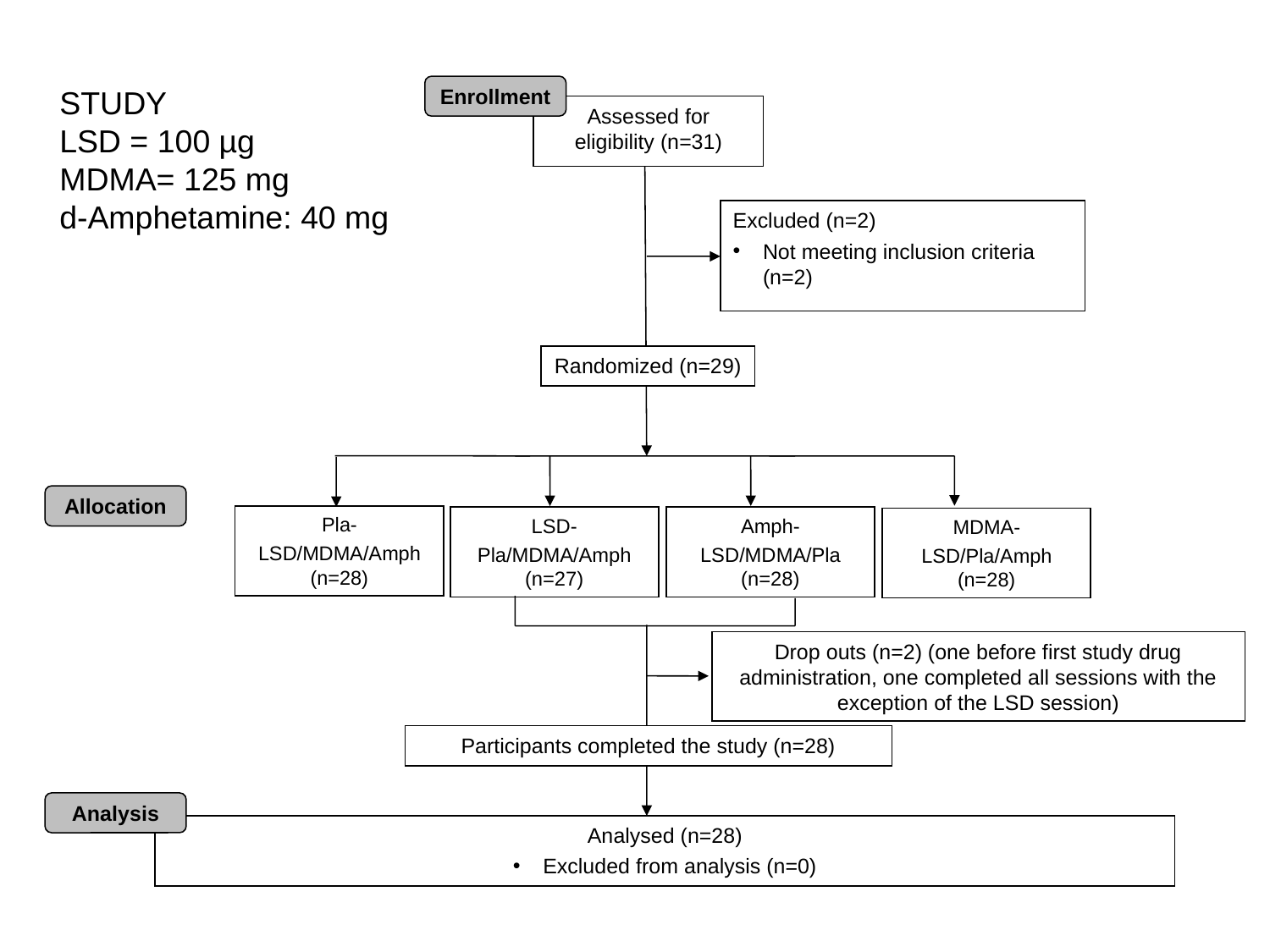

STUDY
LSD = 100 µg
MDMA= 125 mg
d-Amphetamine: 40 mg
Enrollment
# Assessed for eligibility (n=31)
Excluded (n=2)
Not meeting inclusion criteria (n=2)
Randomized (n=29)
Allocation
Pla-
LSD/MDMA/Amph (n=28)
LSD-
Pla/MDMA/Amph (n=27)
Amph-
LSD/MDMA/Pla (n=28)
MDMA-
LSD/Pla/Amph (n=28)
Drop outs (n=2) (one before first study drug administration, one completed all sessions with the exception of the LSD session)
Participants completed the study (n=28)
Analysis
Analysed (n=28)
Excluded from analysis (n=0)
