# Supplementary material for: Distinct acute effects of LSD, MDMA, and d-amphetamine in healthy subjects
Source: Neuropsychopharmacology. 2019 Nov 16;45(3):462–71. doi: 10.1038/s41386-019-0569-3 (PMC6969135; doi:10.1038/s41386-019-0569-3)
Supplement: Supplementary file 2 — Supplementary Data [file 41386_2019_569_MOESM2_ESM.docx]

**Supplementary only data, Holze et al.**

**Figure**

**S1.** Subjective effects of LSD, MDMA, and d-amphetamine on the Addiction Research Center Inventory (ARCI). A, Amphetamine scale; BG, Benzedrine Group; MBG, Morphine-Benzedrine Group; PCAG, Pentobarbital-Chlorpromazine-Alcohol Group; LSD, Lysergic Acid Diethylamide scale. LSD showed significant effects on all subscales indicating hallucinogenic but also mixed stimulant-euphoriant and sedative effects. LSD significantly reduced BG ratings compared with MDMA and d-amphetamine indicating lower stimulant effects (Table S1). Additionally, LSD significantly increased PCAG ratings compared with MDMA and amphetamine indicating more sedating effects. LSD was also the only substance which significantly increased ratings on the LSD scale. MDMA increased ratings on the A and MBG scale indicating amphetamine-type euphoriant effects similar to d-amphetamine. MDMA produced no significant effects on the BG scale compared with placebo and MDMA produced significantly lower effects on the BG group compared with d-amphetamine indicating only mild stimulant effects. D-amphetamine increases scores on the A, BG, and MBG scale consistent with stimulant-type and euphoriant effects. The data are expressed as mean ± SEM. *P<.05, ***P<.001 vs. Placebo. The corresponding statistics are shown in Table S1.

**

Figure S2.** All three substances significantly increased pupil size in the dark and after a light stimulus and reduced pupillary constriction in response to light. MDMA and LSD but not amphetamine significantly reduced the pupillary constriction amplitude in response to light. Additionally, the reduction in the light-induced constriction response was more pronounced after MDMA compared with LSD or d-amphetamine. D-amphetamine showed no effect on pupillary constriction. The data are expressed as mean ± SEM. The corresponding statistics are shown in Table 1.

**

**

**Figure S3.** Plasma concentration *vs*. time profiles of LSD, MDMA, d-amphetamine, the LSD metabolite O-H-LSD, and the MDMA metabolites 3,4‑methylenedioxyamphetamine (MDA) and 4-hydroxy-3-methoxymethamphetamine (HMMA). Peak plasma levels of the substances were reached at 1.6 (1-3.5) h, 3.0 (1.1-5.0) h, and 2.6 (1.0-5.5) h after LSD, MDMA, and d-Amphetamine administration, respectively. The data are expressed as the geometric mean and SEM. The substances were administered at t = 0. Time points are shown relative to the administration of each drug.





**Figure S4.** Endocrine effects of LSD, MDMA, d-amphetamine, and placebo. Only MDMA clearly and significantly increased plasma levels of oxytocin compared with placebo. None of the substances showed an effect on BDNF concentrations in plasma. The data are expressed as mean ± SEM. Statistic values are shown in Table 1.
